# Systematic Review: Sleep Disorders Based on Objective Data in Children and Adolescents Treated for a Brain Tumor

**DOI:** 10.3389/fnins.2022.808398

**Published:** 2022-02-22

**Authors:** Anne Sophie Lind Helligsoe, Kathrine Synne Weile, Line Kenborg, Louise Tram Henriksen, Yasmin Lassen-Ramshad, Ali Amidi, Lisa Maria Wu, Jeanette Falck Winther, Line Pickering, René Mathiasen

**Affiliations:** ^1^Department of Pediatrics and Adolescent Medicine, Aarhus University Hospital, Aarhus, Denmark; ^2^Department of Clinical Medicine, Faculty of Health, Aarhus University, Aarhus, Denmark; ^3^Childhood Cancer Research Group, Danish Cancer Society Research Center, Copenhagen, Denmark; ^4^Danish Centre for Particle Therapy, Aarhus University Hospital, Aarhus, Denmark; ^5^Unit for Psychooncology and Health Psychology, Department of Psychology and Behavioural Sciences, Aarhus University, Aarhus, Denmark; ^6^Department of AIAS, Aarhus Institute of Advanced Studies, Aarhus University, Aarhus, Denmark; ^7^Department of Clinical Neurophysiology, Danish Center for Sleep Medicine, Rigshospitalet, University of Copenhagen, Glostrup, Denmark; ^8^Department of Paediatrics and Adolescent Medicine, The University Hospital Rigshospitalet, Copenhagen, Denmark

**Keywords:** childhood, CNS tumor, sleep, polysomnography, sleep disorder

## Abstract

**Background:**

Tumors of the central nervous system (CNS) are the most common solid childhood malignancy. Over the last decades, treatment developments have strongly contributed to the improved overall 5-year survival rate, which is now approaching 75%. However, children now face significant long-term morbidity with late-effects including sleep disorders that may have detrimental impact on everyday functioning and quality of life. The aims of this study were to (1) describe the symptoms that lead to polysomnographic evaluation; (2) describe the nature of sleep disorders diagnosed in survivors of childhood CNS tumor using polysomnography (PSG); and (3) explore the association between tumor location and diagnosed sleep disorder.

**Methods:**

An extensive literature search following the Preferred Reporting Items for Systematic Review and Meta-Analysis guidelines (PRISMA) was conducted. Inclusion criteria were children and adolescents diagnosed with a CNS tumor age <20 years having a PSG performed after end of tumor treatment. The primary outcome was sleep disorder confirmed by PSG.

**Results:**

Of the 1,658 studies identified, 11 met the inclusion criteria. All the included articles were appraised for quality and included in the analysis. Analyses indicated that sleep disorders commonly occur among childhood CNS tumor survivors. Symptoms prior to referral for PSG were excessive daytime sleepiness (EDS), fatigue, irregular breathing during sleep and snoring. The most common sleep disorders diagnosed were sleep-related breathing disorders (i.e., obstructive sleep apnea) and central disorders of hypersomnolence (i.e., narcolepsy).

**Conclusion:**

Our findings point to the potential benefit of systematically registering sleep disorder symptoms among CNS tumor patients together with tumor type and treatment information, so that at-risk patients can be identified early. Moreover, future rigorous and larger scale controlled observational studies that include possible modifiable confounders of sleep disorders such as fatigue and obesity are warranted.

**Clinical Trial Registration:**

https://www.crd.york.ac.uk/prospero/display_record.php?ID=CRD42021243866, identifier [CRD42021243866].

## Background

Tumors of the central nervous system (CNS) are the most common solid childhood malignancy (Grabas et al., 2020). Over the last decades, treatment developments have contributed to a markedly improved overall 5-year survival rate approaching 75% (Lannering et al., 2009; Gatta et al., 2014; Desandes et al., 2020). However, children now face significant long-term morbidity, including sleep disorders, with detrimental impact on everyday functioning and quality of life (Pickering et al., 2017; Jeon et al., 2021) that may be due to the tumor type, tumor location, or treatment (typically a combination of surgery, chemotherapy, and/or radiotherapy).

Sleep disorders are associated with decreased health-related quality of life in the general pediatric and adolescent population (Hart et al., 2005; Owens, 2014). Sleep disorders, sleep disturbances, and excessive daytime sleepiness (EDS) have been reported in children with cancer and associated with an increased risk of hospitalizations (Mulrooney et al., 2008; Kenborg et al., 2019). Furthermore, sleep disturbances can adversely affect social functioning (Walsh et al., 2020) and impair scholastic achievement (Lahteenmaki et al., 2007). In general, CNS tumor survivors have an increased risk of socioeconomic adverse effects compared with other childhood cancer survivors (Frederiksen et al., 2019).

During the cancer trajectory, sleep disturbances may occur during cancer treatments and the year after (Hinds et al., 2007; Daniel et al., 2017) but it is unclear whether childhood CNS tumor survivors are likely to experience sleep disorders later in life. Such diagnostic information would not only be important for cancer survivors to be aware of, but also to inform treatment options.

Research in childhood cancer survivors has primarily focused on sleep disorders captured by self-report data (Mulrooney et al., 2008). Yet, an increasing number of studies are encouraging the evaluation of sleep disorders using polysomnography (PSG) in childhood CNS tumor survivors in order to better understand the nature of their dysfunction (Kaleyias et al., 2012). Therefore, the present study aimed to systematically review the literature to: (1) describe the symptoms that lead to polysomnographic evaluation in survivors of childhood CNS tumor; (2) describe the nature of sleep disorders diagnosed in this group using PSG; and (3) explore the association between tumor location and diagnosed sleep disorder.

## Materials and Methods

The Preferred Reporting Items for Systematic Review and Meta-Analysis guidelines (PRISMA) were followed (Page et al., 2021). A systematic review protocol was designed, and the research group agreed on the search strategy and *a priori* defined inclusion and exclusion criteria. The study protocol was registered with Prospero (registration number CRD 42021243866).

### Search Strategy

We conducted a comprehensive literature search with no restrictions with respect to language or year of publication including (1) electronic searches of PubMed/MEDLINE, Embase/Ovid, Web of Science, and Cochrane Central Register of Controlled Trials (CENTRAL) and (2) searches of reference lists of identified studies, related reviews, and clinical trial registries (ClinicalTrials.gov registry). Our search strategy consisted of three individual blocks combining sleep, CNS tumor, and children. The search strategy for the MEDLINE search is available in Supplementary Table 1. The search was repeated before submission on November 1^st^, 2021.

### Study Selection, Inclusion, and Exclusion Criteria

Studies were included based on the following inclusion criteria: (a) CNS tumor diagnosis before the age of 20 years; (b) PSG performed after the end of treatment; (c) randomized controlled trial (RCT) or observational study with or without a control group; (d) primary outcomes were symptoms leading to PSG and diagnosed sleep disorders; and (e) the association of tumor location and diagnosed sleep disorder was described. Studies were excluded if only (a) electroencephalography or (b) actigraphy were performed, and if they were (c) case reports, case series or small sample studies in which data were not aggregated. Studies where only some of the patients fulfilled the inclusion criteria were included and data on the included individuals were extracted. Two review authors (ASLH, KSW) independently screened and selected studies based on title and abstract using Covidence. The same reviewers subsequently performed full-text screening and data extraction independently. Non-English studies were translated by a translator to decide whether inclusion criteria were met. Disagreements were resolved with discussion. Duplicates were excluded.

### Data Extraction

For each included study, we recorded the first author's name, year of publication, country, study design, age at diagnosis, age at PSG, time since diagnosis, CNS tumor location, treatment, symptoms leading to PSG, and diagnosed sleep disorder based on the International Classification of Sleep Disorders (Sateia, 2014). Studies were grouped according to study design and diagnosed sleep disorder. Central sleep apnea is characterized by a lack of drive to breathe during sleep and can be influenced by tumor location, pharmacological treatment, and surgery to the area and thus interruption of the neurologic circuit (Eckert et al., 2007). Obstructive sleep apnea is characterized by repeated upper airway collapse during sleep leading to desaturation and thereby disrupted sleep (Jordan et al., 2014).

### Quality Assessment

All studies were appraised for quality by two independent researchers (ASLH, KSW) using the standardized Newcastle-Ottawa Scale (Wells et al., 2021) for assessing risk of bias in observational studies as recommended by the Cochrane Collaboration (Higgins et al., 2017). The scale is based on a star rating system with a maximum of nine stars. Risk of bias was assessed in both case control as well as cohort studies according to three criteria: (1) selection of study groups, (2) comparability of the study and control group, and (3) ascertainment of outcome.

For the criteria “selection of groups,” representativeness of the group, selection, and ascertainment of exposure were assessed. For the criteria “comparability between groups” we defined age as a factor of particular relevance for adjustment, as age affects the diagnostic criteria of sleep disorders made after PSG. For the criteria “ascertainment of outcome,” we defined a threshold for minimum follow-up length after end of treatment of 1 year.

## Results

### Characteristics of Included Studies

**Figure 1:**
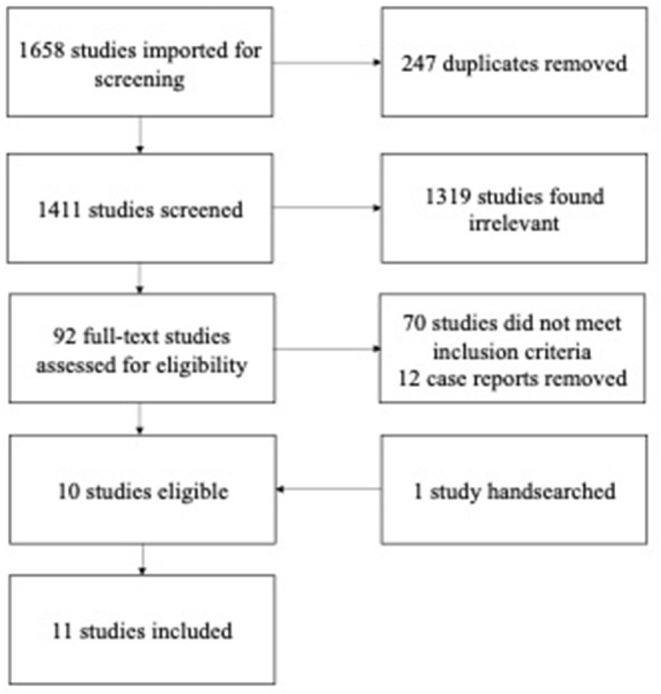
PRISMA flow chart. A total of 1,658 studies were produced. Of these 247 duplicates were removed, and the remaining 1,411 studies were screened. After screening title and abstract 1,319 studies were found irrelevant, as they did not meet eligibility criteria. Seventy studies were excluded as they did not meet inclusion criteria after full-text screening, and 12 were case reports or case series. Finally 11 studies were included.

A total of 1,658 studies were identified and after removal of 247 duplicates, the remaining 1,411 studies were screened using the inclusion and exclusion criteria (Figure 1). After the screening of title and abstract, 1,319 studies did not meet eligibility criteria. Seventy studies were excluded as they did not meet inclusion criteria after full-text screening, and 12 were case reports or case series. One study was hand searched. Eleven studies, published between 1991 and 2021, were included in our analyses. All studies included PSG. One study included polygraphic evaluation of sleep disorders combined with data on respiratory rhythm, and was therefore included in our analyses (Fagioli et al., 1991).

Reasons for exclusion after full-text screening were: Sleep evaluation by cassette recording and not PSG (Palm et al., 1992); ongoing treatment (Chuang et al., 2013; Delrosso et al., 2014); duplicates (Mendez, 1992; Khan et al., 2017); and in one case, the same population had been used twice, thus, we only included the most recent publication (Rosen and Brand, 2011). We did, however, include one study, where only some of the patients met the inclusion criteria (Crowley et al., 2011).

The 11 studies included represented 261 patients with a median age range at diagnosis from 5.6 to 10.1 years, at PSG from 12.4 to 40.5 years, and time from diagnosis from 0.75 year to 10.2 years (Table 1). The studies varied widely by tumor type, age at PSG, sample size and study setting. Tumor treatment consisting of either surgery, chemotherapy, and/or radiation had been completed in all studies except one (Rosen and Brand, 2011) where 25% of the patients were still receiving treatment. Three studies were conference abstracts (Müller et al., 2006; Pilotto et al., 2019; Johnson et al., 2020) and eight studies were full text articles. In four studies (*n* = 39), the patients had been diagnosed with craniopharyngioma (Müller et al., 2006; O'Gorman et al., 2010; Crowley et al., 2011; Manley et al., 2012) and in seven studies (*n* = 222), the population consisted of different CNS tumor diagnoses (Fagioli et al., 1991; Rosen and Brand, 2011; Mandrell et al., 2012; Khan et al., 2017; Pilotto et al., 2019; Johnson et al., 2020; Pickering et al., 2021). In three studies, the patients were diagnosed with both craniopharyngioma and obesity (Müller et al., 2006; O'Gorman et al., 2010; Crowley et al., 2011). In one study (Crowley et al., 2011), only 25% of the study sample (*n* = 7) were diagnosed in childhood and specific sleep disorder data for those individuals could not be separately extracted.

**Table 1:** Characteristics of studies according to country, number of participants, age at diagnosis, age at polysomnography, and quality assessment following the Newcastle-Ottawa Scale.

**Author, Year**	**Country**	**Total number of participants**	**Subjects meeting eligibility criteria**	**Age at diagnosis (median, years)**	**Age at PSG (median, years)**	**Time since diagnosis (years)**	**Quality Assessment (0–9 stars)**
Crowley et al. (2011)	Ireland	28	7	NA	40.5	NA	5
Fagioli et al. (1991)	France	19	19	6.8	NA	NA	5
Johnson et al. (2020) (abstract)	USA	12	12	NA	14	9-72 months	2
Khan et al. (2017)	USA	39	39	10.1	NA	10.2	6
Mandrell et al. (2012)	USA	31	31	7.4	14.3	NA	5
Manley et al. (2012)	USA	19	9 (data on 7)	8	17.5	9	5
Müller et al. (2006)	Germany	115	10	NA	NA	NA	5
O'Gorman et al. (2010)	Canada	15	15	NA	15.5	NA	7
Pickering et al. (2021)	Denmark	61	61	5.6	12.4	5.3	6
Pilotto et al. (2019) (abstract)	Italy	16	12	9.3	12.5	NA	2
Rosen and Brand (2011)	USA	70	48	NA	NA	NA	4

Regarding study design, no RCTs were identified, but five publications included control or comparison groups (O'Gorman et al., 2010; Crowley et al., 2011; Mandrell et al., 2012; Khan et al., 2017; Pickering et al., 2021). Due to heterogeneity and the risk of bias, it was only possible to narratively describe the results with a focus on symptoms that led to PSG, the sleep disorders diagnosed after PSG, and their association with tumor location.

### Quality Assessment

Risk of bias assessment is summarized in Table 2. The quality scale ranged from 0 to 9, where scores below seven was indicative of low quality (Wells et al., 2021). The 11 studies had an average score of 4.7, and only one study (O'Gorman et al., 2010) met criteria for high quality due to its high scores with respect to comparability between groups. In general, studies received the lowest scores with respect to “selection of study groups” and “comparability between groups” and highest scores in “ascertainment of outcome.”

**Table 2:** Quality appraisal of the ten studies included according to Newcastle Ottawa Scale.

**Study author, Year**	**Design**	**Selection 1**	**Selection 2**	**Selection 3**	**Selection 4**	**Comparability 1**	**Exposure/Outcome 1**	**Exposure/Outcome 2**	**Exposure/Outcome 3**	**Total**
Crowley et al. (2011)	Case control	1	0	0	1	1	1	1	0	5
Fagioli et al. (1991)	Cohort	1	NA	1	0	NA	1	1	1	5
Johnson et al. (2020)	Cohort	0	NA	1	0	NA	1	1	0	2
Khan et al. (2017)	Case control	1	1	0	1	2	1	1	1	6
Mandrell et al. (2012)	Cohort	1	NA	1	0	NA	1	0	1	5
Manley et al. (2012)	Cohort	1	NA	1	0	NA	1	1	1	5
Müller et al. (2006)	Cohort	1	NA	1	0	NA	1	0	1	5
O'Gorman et al. (2010)	Case control	1	1	0	1	2	1	1	1	7
Pickering et al. (2021)	Cohort	1	NA	1	0	2	0	1	1	6
Pilotto et al. (2019)	Cohort	1	NA	0	0	NA	1	1	0	2
Rosen and Brand (2011)	Cohort	1	NA	1	0	NA	1	0	0	4
Mean										4,7

*Exposure was related to case-control studies, whereas outcome was related to cohort studies. The questions related in every column refer to the Newcastle Ottawa Scale. Not assessed (NA) is noted when there was no comparison group (Selection 1 = “Adequate case definition/Representativeness of cohort”, Selection 2 = “Representativeness of cases/Selection of non-exposed cohort”, Selection 3 = “Selection of controls/Ascertainment of exposure”, Selection 4 = “Definition of controls/Outcome of interest”, Comparability 1 = “Comparability of cases and controls/cohorts”, Exposure/Outcome 1 = “Ascertainment of exposure/Assessment of outcome”, Exposure/Outcome 2 = “Method of ascertainment/Long enough follow-up”, Exposure/Outcome 3 = “Non-response rate/Adequacy of follow-up”)*.

### Symptoms Leading to Polysomnography

In seven (64%) studies, symptoms prior to referral for PSG were presented (Table 3). The patients reported EDS, fatigue, irregular breathing during sleep, and snoring.

**Table 3:** Characteristics of population, symptoms leading to polysomnography, tumor location, and findings after polysomnography.

**Author**	**Number of participants**	**Population**	**Tumor location**	**Symptoms**	**Findings after PSG**
Fagioli et al. (1991)	19 (18)	Mix of CNS tumors	Cerebellum/4^th^ ventricle (*n* = 18),	NA	Shorter sleeping time and more awakenings compared to controls.
Johnson et al. (2020)	12	Mix of CNS tumors	NA	NA	High risk of sleep wake cycle disorder in early survivorship (9-72 months post treatment). Morning melatonin and biomarker correlates with fatigue 7 clinical sleep disorders, 2 hypersomnia, 1 narcoplepsy.
Khan et al. (2017)	39	Mix of CNS tumors	Cortical (*n* = 4), midline (*n* = 26), paramedian (*n* = 4), posterior fossa (*n* = 5)	Hypersomnia	13 hypersomnia and 26 narcoplepsy without cataplexy. 11/39 abnormal PSG. 37 patients received treatment.
Mandrell et al. (2012)	31	Mix of CNS tumors	Fossa posterior/4^th^ ventricle (*n* = 4+4), sellar/parasellar/hypothalamic (n = 17), optic nerve (*n* = 2), pineal gland (*n* = 1), spinal (*n* = 1), thalamus (*n* = 1), brainstem (*n* = 1)	Excessive daytime sleeping, fatigue, snoring, irregular breathing during sleep	14 obstructive sleep apnea, 4 central sleep apnea, 4 hypersomnia, 3 narcolepsy without cataplexy.
Pickering et al. (2021)	61 (59)	Mix of CNS tumors	Thalamus, hypothalamus, basal forebrain (*n* = 25), fossa posterior (*n* = 16), brain stem (*n* = 5), ventricles (*n* = 3), pineal gland (*n* = 2), optic nerve (*n* = 2), other (*n* = 9)	Sleep disordered breathing, emotional problems, fatigue	51/59 sleep apnea (obstructive, n=29, central, n=5, mixed, n=7), 5/59 narcolepsy, 2/59 NREM parasomnia, 1/59 REM sleep parasomnia.
Pilotto et al. (2019)	16	Mix of CNS tumors	Sub tentorial tumor (*n* = 8)	NA	Increased central apnea index with cerebellum localization.
Rosen and Brand (2011)	48	Mix of CNS tumors	Hypothalamus/brainstem (*n* = 35), posterior fossa (*n* = 7), cortex (*n* = 6)	Sleepiness, fatigue, respiratory insufficiency, snoring	9/14 excessive daytime sleepiness, 5 of them with positive PSG of narcolepsy.
Crowley et al. (2011)	7 (28)	Craniopharyngioma	Suprasellar/Hypothalamic	Somnolence	11/28 obstructive sleep apnea. Somnolence can be due to obstructive sleep apnea in patients with craniopharyngioma.
Manley et al. (2012)	9 (7)	Craniopharyngioma	Suprasellar/Hypothalamic	Day time fatigue, sleep dysfunction	3 obstructive or central sleep apnea, arousal index 11.0, 3 restless legs syndrome. Sleep dysfunction is multifactorial, PSG should be performed more often.
Müller et al. (2006)	10	Craniopharyngioma	Suprasellar/Hypothalamic	Obesity, increased daytime sleepiness	2 sleep related breathing disorder, 4 repeated episodes of SOREM (sleep onset rapid eye movement), 3 hypersomnia, 9 were acutely obese.
O'Gorman et al. (2010)	15	Craniopharyngioma	Suprasellar/Hypothalamic	NA	Obstructive hypopnea apnea index was increased in patients with craniopharyngioma. Sleep disordered breathing is more frequent in patients with craniopharyngioma and obesity compared with BMI matched controls.

### Sleep Disorders Diagnosed After Polysomnography

Findings from the PSGs are listed in Table 3. Seven studies (O'Gorman et al., 2010; Crowley et al., 2011; Rosen and Brand, 2011; Mandrell et al., 2012; Khan et al., 2017; Pilotto et al., 2019; Pickering et al., 2021) reported that the PSG was performed in accordance with guidelines from the American Academy of Sleep Medicine (Iber et al., 2007). In eight studies, specific diagnoses of sleep disorders were noted (Müller et al., 2006; Crowley et al., 2011; Rosen and Brand, 2011; Mandrell et al., 2012; Manley et al., 2012; Khan et al., 2017; Johnson et al., 2020; Pickering et al., 2021). We grouped these in accordance with the International Classification of Sleep Disorders (Sateia, 2014) into: (1) sleep-related breathing disorders, (2) central disorders of hypersomnolence, (3) parasomnias, and (4) sleep-related movement disorders (Table 4), as no other diagnostic categories of sleep disorders were captured in the included studies.

**Table 4:** Sleep disorders classified according to International Classification of Sleep Disorders, Third edition.

**Diagnostic group**	**Sleep disorders**	**Authors**	**Number of patients with sleep disorders/ total of patients included**
Sleep-related breathing disorders	Obstructive, central or mixed sleep apnea	Pickering et al.	51/59
	Obstructive or central sleep apnea	Manley et al.	3/7
	Obstructive sleep apnea	Mandrell et al.	14/31
	Central sleep apnea	Mandrell et al.	4/31
	Central sleep apnea	Pilotto et al.	*n* was unknown
Central disorders of hypersomnolence	Narcolepsy	Pickering et al.	5/61
	Narcolepsy or hypersomnolence	Khan et al.	37/39
	Narcolepsy, hypersomnolence	Mandrell et al.	7/31
	Narcolepsy, hypersomnolence + unknown sleep disorder	Johnson et al.	2+5/12
	Narcolepsy, hypersomnolence	Müller et al.	7/10
Parasomnias	NREM parasomnia	Pickering et al.	2/59
	REM sleep parasomnia		1/59
Sleep-related movement disorders	Restless legs syndrome	Manley et al.	3/7
Delayed sleep phase	Delayed sleep phase syndrome	Rosen et al.	1/48

#### Sleep-Related Breathing Disorders

In four studies (Crowley et al., 2011; Mandrell et al., 2012; Manley et al., 2012; Pickering et al., 2021), 83/129 (64%) patients were diagnosed with sleep-related breathing disorders that included obstructive, central, and mixed sleep apnea (Sateia, 2014). The majority of the patients in the four studies (79/129) had hypothalamic tumor involvement. The largest study of the four (Pickering et al., 2021) reported on 61 patients (respiratory data on 59 patients), of whom 51 of the children were diagnosed with sleep apnea (obstructive *n* = 29, mixed *n* = 7, central *n* = 5). The most common symptoms prior to referral for PSG were EDS, irregular breathing during sleep, snoring, and fatigue. In the second largest study (Mandrell et al., 2012), EDS was confirmed by a short mean sleep latency of 3 min measured by a multiple sleep latency test (MSLT). The MSLT is an objective test of the tendency to fall asleep under controlled conditions (Arand and Bonnet, 2019).

Daytime sleepiness and sleep apnea were assessed in one study with patients with craniopharyngioma (Crowley et al., 2011), and almost 40% (11/28) of the patients with craniopharyngioma presented with EDS and were diagnosed with obstructive sleep apnea. Furthermore, they were treated with continuous positive airway pressure and modafinil and four out of 11 (36%) benefitted from the treatment.

#### Central Disorders of Hypersomnolence

In six studies (Müller et al., 2006; Rosen and Brand, 2011; Mandrell et al., 2012; Khan et al., 2017; Johnson et al., 2020; Pickering et al., 2021), 63 patients complaining of EDS prior to PSG were subsequently diagnosed with either hypersomnia or narcolepsy. In two studies, EDS was assessed by questionnaire with the Epworth Sleepiness Scale (Khan et al., 2017) or the Pediatric Daytime Sleepiness Scale (Pickering et al., 2021). The presence of cataplexy was reported in three studies (Mandrell et al., 2012; Khan et al., 2017; Pickering et al., 2021), and hypocretin levels were not reported in any studies.

Four out of the six studies involved tumors predominantly located in the hypothalamus (Müller et al., 2006; Rosen and Brand, 2011; Mandrell et al., 2012; Pickering et al., 2021), one study with tumors in different locations (Khan et al., 2017), and one with tumors in unknown locations (Johnson et al., 2020).

As expected, the prevalence of EDS and narcolepsy was higher in childhood brain tumor survivors compared with the general population, and in more than 50% of cases (40/77), a diagnosis led to treatment of the sleep disorder (Khan et al., 2017; Pickering et al., 2021). In one study, narcolepsy was diagnosed between 9 and 72 months after cancer treatment (Khan et al., 2017).

Somnolence together with sleep apnea was assessed in one study with patients with craniopharyngioma (Crowley et al., 2011), and as described, almost 40% (11/28) of the patients with craniopharyngioma presented with EDS and were diagnosed with obstructive sleep apnea.

### Tumor Location and Sleep Disorder

Four studies included only patients diagnosed with a suprasellar tumor (*n* = 62), and they were diagnosed with obstructive sleep apnea (*n* = 11), narcolepsy/hypersomnia (*n* = 7), central or obstructive sleep apnea (*n* = 3), and sleep-related breathing disorder (*n* = 2) (Müller et al., 2006; O'Gorman et al., 2010; Crowley et al., 2011; Manley et al., 2012). One study observed an association between central sleep apnea and tumor location in cerebellum suggesting an impact of posterior fossa tumor involvement on sleep and ventilatory control (Pilotto et al., 2019).

Two studies (Fagioli et al., 1991; Pilotto et al., 2019) included patients with cerebellar tumors and found shorter sleeping times and more awakenings compared with normative data. In one study, two patients were diagnosed with non-rapid eye movement (NREM) parasomnias after partial resection of a pilocytic astrocytoma and a ganglioglioma, respectively. One patient was diagnosed with rapid eye movement (REM) sleep parasomnia after partial resection of a diffuse astrocytoma (Pickering et al., 2021). One study reported restless legs syndrome after diagnosis of craniopharyngioma in three out of seven patients (Manley et al., 2012). Two studies reported no specific diagnoses of sleep disorders (Fagioli et al., 1991; O'Gorman et al., 2010).

### Other PSG Findings

One study investigated growth hormone secretion in relation to sleep (Fagioli et al., 1991), and another study reported a higher frequency of sleep disordered breathing in obese patients with craniopharyngiomas than obese controls (O'Gorman et al., 2010).

## Discussion

The present systematic review of 11 published studies indicates that sleep disorders may occur after surviving a childhood CNS tumor. From these studies we found that the symptoms leading to PSG were heterogeneous, and we found no clear association between tumor location and sleep disorder. However, these studies reported a high occurrence of sleep disorders among their patients. By using the diagnostic classification system from the International Classification of Sleep Disorders (Sateia, 2014), the two most common sleep diagnoses captured by the included studies were obstructive sleep apnea and narcolepsy categorized in sleep related breathing disorders and central disorders of hypersomnolence, respectively.

### Symptoms Before PSG

Patients commonly reported EDS, fatigue, irregular breathing during sleep, and snoring prior to PSG. These symptoms were not associated with specific sleep disorders, but fatigue and sleepiness are recognized late effects for childhood cancer survivors and can persist years after end of cancer treatment and lead to psychosocial challenges (Verberne et al., 2012). Central nervous system tumor survivors have an increased risk of EDS compared with children, who have survived other childhood malignancies (van Deuren et al., 2020).

### Sleep Disorder Diagnoses and the Association With Tumor Location

The different types of sleep disorders may be associated with specific tumor locations. Overall, studies reported tumors in a variety of locations including suprasellar, posterior fossa, and brain stem. Indeed, five of the 11 included studies included patients with suprasellar tumors (O'Gorman et al., 2010; Crowley et al., 2011; Mandrell et al., 2012; Manley et al., 2012; Pickering et al., 2021). The most common type of sleep disorder observed in survivors of CNS tumors was sleep apnea. Adenotonsillar hypertrophy is the most important risk factor for obstructive sleep apnea in general pediatric populations (Gislason and Benediktsdóttir, 1995; Dayyat et al., 2009). Obesity increases the risk of obstructive sleep apnea in adolescents and likely also children with specific medical conditions and comorbidities such as children with brain tumors (Jordan et al., 2014). Prior studies have reported that patients with tumor involvement of the hypothalamus may suffer from hypothalamic obesity due to underlying mechanisms causing a combination of increased energy intake and reduced physical activity (Harz et al., 2003; Park et al., 2013).

Narcolepsy was another sleep disorder diagnosed in CNS tumor survivors. In such studies, tumor locations included the hypothalamus, brain stem, or posterior fossa. This association is posited to be due to disruption of sleep- and wakefulness-promoting neural networks caused by the tumor itself or by subsequent treatments such as surgery and/or radiotherapy (Sakuta et al., 2012). Specifically, sleep and wakefulness are regulated by neuronal networks of the thalamus, hypothalamus, basal forebrain and brain stem, and an ascending reticular activation system originating from the upper brainstem and basal forebrain activates the cortex and modulates wakefulness (Saper et al., 2005). Thus, damage to this network or to any of the central nuclei may potentially result in disturbed sleep (Saper, 2013). Manifestation of narcolepsy in CNS tumor survivors due to lack of hypocretin may also relate to the flip-flop switch that mediates transitions between wakefulness and sleep (Saper et al., 2005). The switch is stabilized by orexin produced by neurons located in the lateral hypothalamus. A deficiency of orexin, as seen in patients with narcolepsy type 1, causes undesired switches between sleep and wakefulness resulting in sleep attacks and fragmented sleep pattern. Furthermore, orexin suppresses REM sleep which explains the REM sleep dissociation events observed in these patients. Among patients with CNS tumors, numerous studies have reported secondary/comorbid narcolepsy in those patients with tumors involving the hypothalamic area or close to the third ventricle (Kanbayashi et al., 2006; Sakuta et al., 2012; Madan et al., 2021). Mogavero et al. propose that a diagnosis of neurodegenerative disease may decrease the risk of cancer i.e., the inverse comorbidity mechanism (Mogavero et al., 2020, 2021). Narcolepsy type 1 results from an autoimmune destruction of the orexin producing neurons in the lateral hypothalamus. An interconnection between narcolepsy and cancer, based on genetic and immunological factors, may therefore be hypothesized. As orexin deficiency is the pathological feature in narcolepsy type 1, orexin may play a role in some tumor types. Numerous studies have reported on secondary/comorbid narcolepsy in patients with brain tumors, which may be due to an involvement of the orexin producing neurons or their projections (Marcus et al., 2002; Mandrell et al., 2012). In children with brain tumors, comorbid narcolepsy was observed in 8% and sleep apnea in 86% (Pickering et al., 2021). In comparison, the prevalence of narcolepsy type 1 in the general population is about 0.03% (Longstreth et al., 2007) and sleep apnea 1–10% (Tsukada et al., 2018). On that note, a brain tumor diagnosis does not seem to provide protection against sleep disorders.

Where the cerebellum has a key role in central respiratory control (Stoodley et al., 2012), the brainstem plays an important modulating role (Feldman and Del Negro, 2006). In our study, few patients with brainstem tumors were included, but they were often diagnosed with either hypersomnolence or narcoplepsy (Rosen and Brand, 2011; Mandrell et al., 2012; Pickering et al., 2021). Other studies have described sleep disordered breathing associated with brainstem tumors (Osanai et al., 1994; Ito et al., 1996).

In this review, one study reported sleep-related movement disorder in three out of seven patients with restless legs syndrome (Manley et al., 2012). Another study reported of periodic leg movement, which did not interfere with sleep and thus not classified as a sleep disorder (Khan et al., 2017). Only a few patients in this review were diagnosed with parasomnias, although it has been reported in case reports previously (Cordani et al., 2021). Moreover, none of the 11 studies included described insomnia among their patients. Activity of the ventrolateral preoptic nucleus located in the anterior hypothalamus is central to sleep promotion and it has been reported that involvement of the area results in insomnia (Nofzinger et al., 2004), and insomnia is prevalent both in childhood CNS tumor survivors as well as in the general population (Morin et al., 2011). However, insomnia may be underreported in this review, due to our inclusion criteria of PSG, as insomnia is typically captured using patient-reported outcome measures (Ohayon and Reynolds, 2009).

Patients in our systematic review were all diagnosed before the age of 20 years but underwent PSG between 12.4 and 40.5 years of age. The study population is therefore heterogenous with respect to age, and different sleep disorders are prevalent in different age groups just as one sleep disorder may change across the life span (Lividini et al., 2021). Importantly, PSG was scored in relation to age (Berry et al., 2012). Sleep architecture and the amount of sleep needed changes from childhood into adulthood (Kahn et al., 1996; Quan et al., 2003; McLaughlin Crabtree and Williams, 2009; Owens and Weiss, 2017). In adolescence, altered psychosocial life and maturing of biological processes regulating sleep/wake systems can alter the homeostatic process that works together with the circadian timing system, and this maturation of the biological processes leads to a lower total sleep time in adulthood compared to childhood (Carskadon et al., 2004). Furthermore, delayed sleep phase in adolescents can be debilitating and complicate the diagnosis of other sleep disorders (Thorpy et al., 1988).

### Strengths and Limitations

To the best of our knowledge, this is the first systematic review undertaken of studies on PSG-diagnosed sleep disorders in childhood CNS tumor survivors. In addition, we examined associations between sleep disorders and tumor location, the results of which can inform researchers, health care providers and patients about this important late effect during the cancer trajectory.

This review highlights a number of limitations across research in this area. First, all but one of the studies were rated with a high risk of bias. The studies were heterogenous when comparing study population and design, and only five studies included a control group. Second, the included studies were published between the years 1991 and 2021, during which cancer treatment improved significantly, further reducing comparability between studies. In addition, several potential confounders such as fatigue and obesity were also not investigated in depth (Mulrooney et al., 2008; Crabtree et al., 2010). Third, some studies only conducted PSG on selected patients with specific symptoms, such as EDS, while other studies conducted PSG on all included patients independently of symptoms of a sleep disorder. Fifth, PSG evaluations of children can be limited by the lack of normative data for children under 18 years (Ng and Chan, 2013). Lastly, the majority of sleep disorders in children are diagnosed based on medical history combined with validated sleep questionnaires. Polysomnography is mandatory only when diagnosing obstructive sleep apnea, PSG combined with MSLT for narcolepsy and actigraphy for diagnosing circadian rhythm disorders. Therefore, children diagnosed with either questionnaires or actigraphy were not included in this review.

It is also important to note that we did not consider the effects of specific treatments on the diagnosis of sleep disorders, and we know that treatment alone can have an effect on the developing brain (Mogavero et al., 2020). Furthermore, patient reported outcome measures may better capture other sleep disorders, such as insomnia that commonly occur in cancer survivors (Merz and Tomfohr-Madsen, 2018; Tonning Olsson et al., 2020). Thus, an overall evaluation of the frequency of the full range of sleep disorders in childhood CNS survivors could not be determined from this review.

### Conclusion, Research Implications, and Perspectives

In conclusion, the identification of symptoms of a PSG-diagnosed sleep disorder and potential associations with tumor location can provide important information regarding this important late effect that can impair quality of life for childhood CNS tumor survivors. In the future, it will benefit health care practitioners and patients to systematically register sleep disorder symptoms together with tumor type and treatment information in patients, with an awareness of known discrepancies between subjective and objective sleep evaluations (Jackowska et al., 2011; Lubas et al., 2021). By doing so, clinicians can better identify patients who are likely to need referral for PSG. Furthermore, more rigorous and larger scale controlled observational studies are warranted focusing on possible modifiable confounders of sleep disorders such as fatigue and obesity. Overall, capturing sleep disorder symptoms would inform the development of interventions for affected children, with the ultimate goal of improving social functioning, educational attainment and health related quality of life in childhood CNS tumor survivors.

## Data Availability Statement

The original contributions presented in the study are included in the article/Supplementary Material, further inquiries can be directed to the corresponding author/s.

## Author Contributions

AH designed the study, collected, analyzed, and interpreted the data, wrote and edited the manuscript. KW designed the study, analyzed and interpreted the data, and critically reviewed the manuscript. LK, LH, YL-R, AA, LW, JW, LP, and RM designed the study, interpreted the data, and critically reviewed the manuscript. All authors contributed to the article and approved the submitted version.

## Funding

AH's work was supported by the Danish Childhood Cancer Foundation, Lizzy and Mogens Staal Foundation, Dagmar Marshall Foundation, Tømrermester Jørgen Holm & Hustru Elisa F. Hansen Foundation, Health Research Foundation of Central Denmark Region, and Aarhus University. LW's effort was supported by the European Union's Horizon 2020 Research and Innovation Programme under the Marie Sklodowska-Curie Grant Agreement No. 754513 and the Aarhus University Research Foundation. The funders had no role in designing or conducting the study.

## Conflict of Interest

The authors declare that the research was conducted in the absence of any commercial or financial relationships that could be construed as a potential conflict of interest.

## Publisher's Note

All claims expressed in this article are solely those of the authors and do not necessarily represent those of their affiliated organizations, or those of the publisher, the editors and the reviewers. Any product that may be evaluated in this article, or claim that may be made by its manufacturer, is not guaranteed or endorsed by the publisher.
