# Supplementary material for: Systematic Review: Sleep Disorders Based on Objective Data in Children and Adolescents Treated for a Brain Tumor
Source: Front Neurosci. 2022 Feb 22;16:808398. doi: 10.3389/fnins.2022.808398 (PMC8902496; doi:10.3389/fnins.2022.808398)
Supplement: Supplementary file 1 [file Table_1.docx]

| Supplementary Information Table S1 | | |
| --- | --- | --- |
| Search name | Search query | Type of search |
| Block 1: Sleep |  |  |
| 1 | ("Sleep"[Mesh]) OR ("Fatigue"[Mesh]) OR ("Sleep Wake Disorders"[Mesh]) OR ("Sleep disorder*" OR" Hypersomnia" OR "hypersomnolence" OR "Parasomnia" OR "sleep*") OR ("REM sleep behavior disorder"[Text Word] OR "Periodic limb movement during sleep"[Text Word] OR "obstructive sleep apnea"[Text Word] OR "Sleep apnea"[Text Word] OR "Circadian rhythm sleep-wake disorder"[Text Word] OR "Sleep-related movement disorder"[Text Word] OR "Narcolepsy"[Text Word] OR "Insomnia"[Text Word] OR "Sleep-related breathing disorder*"[Text Word] OR "Sleep apnea"[Text Word] OR "Sleep-related hypoventilation disorder*"[Text Word] OR "Excessive daytime sleepiness"[Text Word] OR "Restless legs syndrome"[Text Word] OR "Kline-Levin Syndrome"[Text Word] OR "Sleep disruption"[Text Word] OR "wakefulness"[Text Word] OR "waking"[Text Word])) | MeSH terms and keywords |
| Block 2: Children |  |  |
| 2 | (Infan* OR newborn* OR new-born* OR perinat* OR neonat* OR baby OR baby* OR babies OR toddler* OR minors OR minors* OR boy OR boys OR boyfriend OR boyhood OR girl* OR kid OR kids OR child OR child* OR children* OR schoolchild* OR schoolchild OR school child[tiab] OR school child*[tiab] OR adolescen* OR juvenil* OR youth* OR teen* OR under*age* OR pubescen* OR pediatrics[mh] OR pediatric* OR paediatric* OR peadiatric* OR school[tiab] OR school*[tiab] OR prematur* OR preterm*) | MeSH terms and keywords |
| Block 3: CNS tumor |  |  |
| 3 | ("central nervous system neoplasms"[MeSH Terms] OR ("central"[All Fields] AND "nervous"[All Fields] AND "system"[All Fields] AND "neoplasms"[All Fields]) OR "central nervous system neoplasms"[All Fields] OR ("cns"[All Fields] AND "tumor"[All Fields]) OR "cns tumor"[All Fields]) AND ("brain tumour"[All Fields] OR "brain neoplasms"[MeSH Terms] OR ("brain"[All Fields] AND "neoplasms"[All Fields]) OR "brain neoplasms"[All Fields] OR ("brain"[All Fields] AND "tumor"[All Fields]) OR "brain tumor"[All Fields]) | MeSH terms and keywords |
| Final search: 1 AND 2 AND 3 |  |  |
|  | | |
